# FLASH radiotherapy as an emerging paradigm in radioimmunotherapy: biological rationale, preclinical evidence, and translational roadmap

**DOI:** 10.3389/fimmu.2026.1869222

**Published:** 2026-08-04

**Authors:** Dongliang Li, Xiaofeng Wang, Shun Lu, Lintao Li, Yue Su, Xianliang Wang, Yongjie Li, Jinyi Lang, Jie Zhou

**Affiliations:** 1 Department of Radiation Oncology, Precision Radiation in Oncology Key Laboratory of Sichuan Province, Sichuan Clinical Research Center for Cancer, Sichuan Cancer Hospital & Institute, Sichuan Cancer Center, School of Medicine, University of Electronic Science and Technology of China, Chengdu, China; 2 School of Life Science and Technology, University of Electronic Science and Technology of China, Chengdu, Sichuan, China

**Keywords:** cancer, flash effect, FLASH-RT, immunotherapy, radiotherapy

## Abstract

Ultra-high dose rate radiotherapy (FLASH-RT) has emerged as a promising technology in recent years. Unlike conventional radiotherapy (CONV-RT), FLASH-RT delivers radiation at an ultra-high dose rate over a very short duration. This approach not only greatly shortens the treatment time, but also exhibits the “FLASH effect”, which significantly spares healthy tissues while maintaining antitumor efficacy comparable to that of CONV-RT. Radiotherapy ignites immunity, fueling the radioimmunotherapy boom. However, even the most advanced radiotherapy techniques inevitably expose healthy tissue, immune organs, vasculature, and circulating or infiltrated lymphocytes to radiation-induced toxicity, limiting the synergistic effect of radioimmunotherapy. Notably, FLASH-RT offers a considerable alternative by protecting the immune system, converting the immunosuppressive tumor microenvironment (TME) into a moderately immune-infiltrated TME, and reducing immunosuppressive responses, thereby enhancing antitumor immunity. A growing number of studies have demonstrated that this combination shows synergistic antitumor effects even in drug-resistant tumor models. Despite these encouraging findings, the combination of FLASH-RT with immunotherapy remains in its early stages and has yet to reach clinical implementation. In this review, we present the current status, underlying mechanisms, and future prospects of FLASH-RT, with a particular focus on its immunomodulatory abilities and potential as a future platform for cancer radioimmunotherapy.

## 1 Background

Radiotherapy (RT) is a cornerstone of cancer treatment, utilized in over 50% of patients (1). However, even the most advanced radiotherapy with advancements in computer-assisted techniques, dosimetric and physical parameters, and radiation modality cannot entirely spare tumor-adjacent healthy tissue or healthy tissue along the radiation path from radiation exposure (2, 3). Recently, an emerging radiation technique called ultra-high dose rate radiotherapy (FLASH-RT) has attracted much attention. FLASH-RT administers doses at an ultra-high dose rate (≥40 Gy/s). This approach not only greatly shortens the treatment time, but also exerts the FLASH effect, which spares healthy tissue while maintaining comparable antitumor efficacy to CONV-RT (4). Since its first clinical application in 2018 (5), FLASH-RT has been considered as a promising technological advancement in radiation oncology, though its clinical efficacy and broad applicability remain under active investigation. Mechanistically, FLASH-RT was originally hypothesized to create a protective hypoxic environment for healthy tissue by rapidly consuming oxygen, thereby sparing healthy tissue (6). Subsequent studies have suggested that multiple pathways and molecules, including those related to DNA damage and repair, mitochondrial and iron metabolism, vascular effects, and immune responses also contribute to the FLASH effect. In-depth investigations into the underlying mechanisms are actively ongoing.

Currently, RT combined with immunotherapy is a promising strategy with diverse applications in clinical settings. Compared to RT or immunotherapy alone, the combination strategy achieves better therapeutic efficacy by remodeling the immunosuppressive tumor microenvironment (TME) to boost immune responses (7). Nevertheless, inevitably radiation exposure to healthy tissue restricts the clinical potency of radioimmunotherapy (RIT) (8). Hence, combining precise RT and immunotherapy represents a focal point of the next wave of RIT, which aims to reduce the radiation-induced toxicity to healthy tissue while preserving or enhancing antitumor efficacy (9). Notably, FLASH-RT offers a potential solution to these limitations. It is a viable candidate for integration with immunotherapy owing to its lower toxicity to healthy tissues, the immune system, and vasculature, combined with its ability to elicit robust antitumor immune responses. Emerging evidence indicates that FLASH-RT combined with anti-PD-1/L1, anti-CTLA-4, CAR-T, and immune-regulated drugs exhibits synergistic efficacy even in ICI- or radio-resistant tumors (10–14). Despite these encouraging outcomes, the combination of FLASH-RT and immunotherapy remained limited by the immaturity of FLASH-RT devices, unclear biological mechanisms, and an inadequate theoretical framework for this combination.

Therefore, this review presents the current status, challenges, and prospects of FLASH-RT. Subsequently, we discuss the current views on the mechanisms underlying FLASH-RT, with a focus on how it remodels the TME in a manner distinct from CONV-RT. Moreover, we highlight the prospects of combining FLASH-RT with immunotherapy, and its potential to overcome the clinical limitations of CONV-RT, with the aim of reducing radiation-induced toxicity while maximizing therapeutic efficacy.

## 2 Current status of FLASH-RT

### 2.1 Dosimetric and physical parameters of FLASH-RT

Compared with CONV-RT (0.01-0.4 Gy/s), FLASH-RT delivers radiation at ultra-high dose rates (exceeding 40 Gy/s). This physical characteristic drastically shortens treatment time, thereby protecting healthy tissue and mitigating adverse effects induced by organ motion. However, the physical and dosimetric parameters that trigger the FLASH effect remain to be systematically characterized and standardized across preclinical and clinical platforms.

Both total dose and dose rate shape the FLASH effect. Preliminary data indicate that FLASH-RT requires approximately 10% higher irradiation dose for intestine tissue and 50% higher dose in skin to reach the same acute- or late-phase toxicity comparable to those from CONV-RT (15–18), which is quantified by the dose-modifying factor (DMF) or FLASH factor. However, different organs require a varying minimum single dose to trigger the FLASH effect (19), and a sufficiently high dose can induce loss of the FLASH effect (20). As summarized from *in vivo* experimental dataset (**Table 1**), approximate dose thresholds for the FLASH effect vary by organ: 8–10 Gy for the brain, 17–20 Gy for the lung, 10–16 Gy for the gastrointestinal tract, and 20–45 Gy for the skin. Regarding dose rate, three studies did not observe the FLASH effect with electron or photon beams when the dose rate was between 30 and 40 Gy/s (21–23), while one study observed it at a dose rate of 30 Gy/s with proton beams (24). This phenomenon probably stems from the combined effects of proton-specific Bragg peak properties, including localized dose-rate amplification and high linear energy transfer, as well as technical variables such as instantaneous and average dose rates and irradiated volume. These observations suggest that the FLASH effect is governed by a biologically effective thresholds of total dose and dose rate, which are highly dependent on radiation beam type, tissue context, and experimental models. Future work should prioritize establishing a standardized definition of biologically effective thresholds to facilitate the clinical translation of diverse radiation modalities. Additionally, precise optimization of dose and dose rate parameters is also critical to maximize the therapeutic benefits of the FLASH effect in subsequent investigations.

**Table 1:** Potential mechanisms of FLASH-RT in *in vivo* experiments.

Irradiated target	Model	Radiation dose		Radiation modality	Main phenomena	Potential factors related to the FLASH effect	Ref
		Mean dose rate (Gy/s)	Total dose (Gy × Fraction)				
Brain	Mice	5.6×10^6^	10 × 2f	Protons	Hypo-fractionated FLASH-RT showed long-term neurological tissue and cognitive sparing effects	Less damage to microvasculature, less persistent neuroinflammation caused by CD68+ microglia	(165)
Brain; Glioblastoma	Mice	>40	10	Protons	FLASH-RT reduced irradiated toxicity to the brain and had a similar antitumor effect with CONV-RT	Less DNA damage (γH2AX)	(99)
Brain	Mice	5.6×10^6^	10 × 3f	Electrons	Hypo-fractionated FLASH-RT showed long-term cognitive protection and less inflammatory responses	Less neuroinflammation induced by CD68^+^ microglia and TLR4	(26)
Brain	Mice	>100	10	Electrons	FLASH-RT protected cognitive function and neuronal structure	Less production of local H_2_O_2_ (ROS) and less neuroinflammation by CD68^+^ microglia	(60)
Brain	Mice	200-300	30	Electrons	FLASH-RT protected cognitive function and neuronal structure	Lower release of neuroinflammatory cytokines (IL-6, TNF-α, IL-1β)	(146)
Brain	Mice	4.4×10^6^	8	Electrons	Single and fractionated FLASH-RT protected cognitive function and newborn neurons	Less dysregulation of hormonal (growth hormone) and less neuroinflammation by CD68^+^ microglia	(84)
Brain	Mice	5.6×10^6^ 2500	10, 25	Electrons	FLASH-RT protected the microvasculature	Less release of eNOS, and stable expression of tight junction protein (occluding, claudin-5)	(61)
Brain	Mice	120	9.6	Protons	FLASH-RT protected the microvasculature and neural function	Less DNA damage (γH2AX), and inflammatory responses (CD68^+^ microglia and macrophages, HMGB-1)	(83)
Brain	Mice	230	10	Electrons	FLASH-RT protected neural function and the gut microbiota under whole-brain irradiation	Upregulation of probiotics which contribute to the regeneration of neurons	(179)
Brain; Glioma	Rat	257	25	Protons	FLASH-RT protected cognitive function and had similar immune activation as CONV-RT	Similar ability to activate the immune system (increased T cells, B cells and NK cells, higher expression of MHC-II)	(127)
Brain; Glioblastoma	Mice	>100	10, 7 × 2f, 3 × 10f	Electrons	FLASH-RT protected cognitive function, hypo-fractionated protocols could maximize the FLASH effect	Less production of ROS in healthy tissue, less neuroinflammation	(25)
Brain Glioma	Mice	80–135	10	Protons	FLASH-RT enhanced antitumor immune responses and the efficacy when combined with CAR-T	Increasing number of CD8+ T cells, M1 and the ratio of CD8+/CD4+, decreased M2 and expression of Arg1, led by PPARγ	(13)
Lung; Intestine; Lung cancer	Mice	>2000	8, 10; 15, 20	Electrons	FLASH-RT protected lung and intestine tissue and undiminished antitumor effect	Similar level of ferroptosis in tumor tissue, low level of ferroptosis in healthy tissue induced by low lipid peroxidation	(95)
Lung; Breast cancer; Head and neck cancer	Mice	40	15–30	Electrons	FLASH-RT protected the lungs, blood vessels, and the bronchi with similar antitumor ability	Less fibrosis led by TGF-β/SMAD and less apoptosis (caspase-3, TUNEL)	(4)
Lung	Mice	–	5.2, 17	Electrons	FLASH-RT presented less persistent DNA damage and spared lung progenitor cells	Less 53bp1 related DNA damage level, less inflammatory (TGF-β) and senescent response (SA-βgal)	(72)
Lung; Heart	Mice	340	17	Electrons	FLASH-RT reduced fibrosis in lung and heart, reduced apoptosis of immune cells	Downregulation of Chk1-STAT3 pathway accelerate recovery of immune cells, decreased Tregs and PD-1/L1	(122)
Lung cancer	Mice	60	18	Protons	FLASH-RT was more efficient for tumor control than CONV-RT	Increased CD8^+^ T cells and M1, decreased Tregs, M2 and PD-1/L1	(129)
Lung cancer; Glioma; Glioblastoma; Head and neck cancer	Mice	>2000	20, 6 × 2f, 8 × 3f	Electrons	FLASH-RT induced equivalent antitumor and long-lasting immunological memory responses as CONV-RT, even in an extreme immunodeficient context	Similar infiltration of immune cells and expression of cytokines. Lower expression of TGF-β	(28)
Lung cancer	Mice	40	18	Protons	FLASH-RT induced more efficient tumor eradication	Increased CD3^+^, CD4^+^, CD8^+^ T cells recruitment	(125)
Lung cancer	Mice	352	15	Electrons	FLASH-RT induced more damage to tumor tissue	Increased ROS level induced by MLC, promoted CD8+ T cells recruitment, and less DNA (γH2AX) damage	(126)
Lung	Mice	>40	17.6	Electrons	FLASH-RT protected lung tissue and promoted its repair	Less infiltration of inflammatory immune cells, short-term activation of CD4+ Th cells, increased level of TGF-β and EMT genes	(139)
Whole Abdomen; Pancreatic cancer	Mice	125	10	Electrons	FLASH-RT induced similar immune responses, reducing spleen and intestine damage	The rate of CD8+/CD4+ and CD8+/CD3+ increased, CD4+/CD3+ decreased. Less inflammatory responses in healthy tissue	(128)
Whole Abdomen	Mice	2–6 × 10^6^	7.5–12.5	Electrons	FLASH-RT spared intestine and gut microbiota	Less toxicity to gut microbiota	(18)
Whole Abdomen; Colon cancer	Mice	110-120	13, 5× 5f	X-rays	FLASH-RT spared the intestine and had a similar antitumor immune response as CONV-RT (similar abscopal effect)	Less activation of cGAS-STING pathway led by pyroptosis (GSDME) in normal tissue	(10)
Whole Abdomen	Mice	150	10, 15	X-rays	FLASH-RT protected the intestinal function and structure	Less lipid damage, less long-term inflammation induced by TNF-α and IL-6	(94)
T cell Lymphoblastic leukemia	Mice	210	4	Electrons	FLASH-RT was more efficient than CONV-RT in reducing T cell lymphoblastic leukemia, while induce less human blood stem cells damage	Less toxicity to the circulating immune system	(120)
Skin	Mice	130	25–30	Protons	FLASH-RT induced long-term protection in the skin	Low concentration of oxygen	(66)
Skin; Muscle; Osteosarcoma	Mice	>100	20	12C-ions	FLASH-RT reduced damage to muscle and skin tissue and undiminished antitumor immune responses	Increasing level of CD8^+^ T cells, and decreased Tregs	(130)
Oral; Head and neck cancer	Mice	218	14–18, 8 × 3f	Protons	FLASH-RT reduced toxicity to oral function and undiminished antitumor effect	Less inflammatory responses induced by TGF-β	(27)
Head and neck cancer	Mice	179	4, 6	X-rays	FLASH-RT inhibited the proliferation and invasion of radioresistant cancer cells, promoted apoptosis and caused DNA damage	Higher activation of CD8+ T cells which stimulated M1 through IFN-γ signaling. M1 secreted CXCL9 to reactivate CD8+ T cells in turn. Increased damage to tumor DNA and enhanced immune response (abscopal effect)	(12)
Ear	Mice	9.3, 930	23, 33	Protons	FLASH-RT reduced ear toxicity and irradiated blood volume, and no obvious difference in TGF-β1, IL-1α, IL-1β and TNF-α	Less irradiated blood volume	(159)
Whole abdomen; Ovarian cancer	Mice	216	14–16	Electrons	FLASH-RT reduced lethal intestinal injury	Less toxicity to circulating immune cells, less DNA damage (γH2AX) induced by ROS. Survival rate of stem cells	(98)
Glioma	Mice	90	15	Electrons	FLASH-RT has a similar antitumor effect to CONV-RT but better immune responses	Short-term and late inflammatory responses induced by IFN-I, stronger immunological memory	(142)
Glioblastoma	Rat	66	12.5 × 2f, 8 × 2f	Protons	FLASH-RT has a similar antitumor effect to CONV-RT	Long-term immunological memory	(29)
Head and neck cancer; Lung cancer; Glioblastoma	Mice	100	20	Electrons	FLASH-RT controlled tumor even in an acute hypoxic environment	Stronger cell-cycle arrest and translational suppression under hypoxia conditions. Metabolic reprogramming towards downregulated OXPHOS and upregulated glycolysis (HIF-1)	(71)
Muscle; Osteosarcoma	Mice	100	18	12C-ions	FLASH-RT protected muscle tissue and reduced lung metastasis	Enhanced immune responses (abscopal effect)	(82)
Skin; Muscle; Head and neck cancer	Mice	57,115	15, 35	Protons	FLASH-RT induced less toxicity to skin and leg contracture and unattenuated antitumor efficacy	Downregulated of TGF-β, CXCL-1 and G-CSF, upregulated expression of GM-CSF	(138)
Skin; Muscle; Bone; Osteosarcoma	Mice Dog	69–124	30–45	Protons	FLASH-RT reduced skin, muscle, bone, and lymphatic system damage, upregulated tissue and vascular repair pathways	Downregulated TGF-β and damages to stem cells	(85)
Zebrafish embryo	Fish	300 286/2.5 × 10^5^	32	Protons Electrons	FLASH-RT reduced irradiated toxicity to the zebrafish embryo	Low concentration of oxygen	(34)
Zebrafish embryo	Fish	1 × 10^5^	26	Electrons	FLASH-RT reduced the number and severity of morphologic malformations	Reduction of the partial oxygen level of irradiated tissue	(63)

Fractionation also impacts the FLASH effect. Most studies currently deliver only a single high dose to reduce the potential adverse effects induced by multiple fractions. Emerging evidence indicates that hypofractionated FLASH-RT protects the brain (7 Gy × 2f, 10 Gy × 3f) (25, 26), oral cavity (8 Gy × 3f) (27), and intestine (5 Gy × 5f) (10). Meanwhile, the antitumor effect of FLASH-RT was found to be nearly the same as CONV-RT in lung cancer (6 Gy × 2f, 8 Gy × 3f) (28), head and neck cancer (8 Gy × 3f) (27), and glioblastoma (8 Gy × 2f, 12.5 Gy × 2f) (29). However, recent studies have proposed that in skin and intestine, more than 2 fractions attenuate the FLASH-RT protective effect, with the FLASH effect disappearing under 10 fractions (30, 31). These results may be attributed to the reduced average dose rates associated with multiple fractions, tissue reoxygenation, and the damage to tissue repair processes by subsequent irradiation. Further studies are needed to carefully optimize fractionation regimens to preserve the FLASH effect. Interestingly, reirradiation with hypofractionated FLASH not only spared skin (15 Gy + 11 Gy × 3f), bone (15 Gy + 11 Gy × 3f), and intestine (12 Gy + 6.4 Gy × 3f), but also enhanced antitumor efficacy (32).

Importantly, pulse structure, pulse number, dose per pulse (pulse width) and pulse interval (pulse frequency) also influence the occurrence of the FLASH effect. Current studies have indicated that high average dose rate (>100 Gy/s) (33, 34), high dose per pulse (> 1 Gy) (35, 36), small pulse number (1-2 pulses) and short pulse interval (<0.7 s) (18) may be associated with enhanced healthy tissue sparing effects.

FLASH-RT can be administered using various irradiation beams, including electrons, protons, X-rays, and heavy ions, which all induce the FLASH effect. However, the roles of individual particles and their linear energy transfer (LET) remain unclear. Most studies use low-energy electron FLASH (e-FLASH) due to its simplicity in achieving sufficient dose rates. However, e-FLASH is primarily suitable for treating superficial tumors, as energy straggling and electron scattering in tissue limit its deeper applicability (37). Very high energy electrons (VHEE) with ultra-high dose rate can be applied to treat deep-seated tumors, but are limited by unstable dose delivery and the lack of medical-grade accelerator. In contrast, proton FLASH (p-FLASH) has been widely applied to deeper tumors and is the most promising type of FLASH-RT. This is due to the Bragg peak effect, where protons deposit their energy at the end of their range with minimal dose delivered in front of the target volume and none behind it (38). Thus, p-FLASH ensures precise targeting of tumor tissue while minimizing potential side effects on healthy tissue. X-rays induced by linear electron accelerators are the most conventional clinical treatment for deeper tumors (39), which has great potential for application in future FLASH-RT. However, X-ray FLASH (x-FLASH) lags behind e-FLASH and p-FLASH due to inefficiencies in converting electrons to X-rays and challenges related to heat deposition in the target (40). Heavy ions, such as carbon and helium offer therapeutic potential owing to the high LET that can exert superior radiobiological effects, but are constrained by the high costs of gantry technology (41).

Overall, several dosimetric and physical parameters, such as total dose, dose rate, fractionation, and pulse structure, have been widely studied to determine the conditions under which the FLASH effect occurs. However, the thresholds for these parameters vary across organs and experimental models. Future studies should systematically investigate the parameters influencing the FLASH effect to establish more refined and standardized thresholds. p-FLASH and x-FLASH hold promise for treating deep-seated tumors in future clinical applications, although realizing their full potential remains challenging. Conversely, e-FLASH, being easier to implement, remains instrumental in exploring the radiobiological mechanisms underlying the FLASH effect and treating superficial tumors.

### 2.2 Current challenges of FLASH-RT clinical translation

Currently, FLASH-RT lacks a precise and feasible dose delivery system, confirmed radiobiological mechanisms, and phase III clinical trials to prove its safety and efficacy. The foremost challenge in FLASH-RT is achieving highly stable and precise ultra-high dose rate radiation, which requires subsecond-scale delivery and a sufficient intensity modulation system. Multiport irradiation simultaneously from different directions toward the targeted tumor is also required, which may reduce the repeated radiation-induced toxicity to healthy tissue (42), as intermittent irradiation of FLASH-RT may attenuate the FLASH effect (30). To avoid incorrect dose delivery in such a short treatment time under FLASH-RT, especially in the treatment of pelvic or abdominal tumors with high motion (43), real-time delivery and motion monitoring, and suspension of equipment for the wrong dose are crucial. Additionally, optimum parameters for FLASH-RT have not yet been established, whereas CONV-RT already has a strict national or international standards regulating its parameters with active onboard dosimetric monitoring and extensive clinical trials (44, 45). Furthermore, the poor reproducibility of the FLASH-RT significantly hinders its clinical translation (46). Even when the dose rate and total dose remain constant, treatment outcomes can vary substantially, sometimes yielding contradictory results. These variations may be attributed to differences in irradiated organs, animal models and dosimetric parameters (47). Additionally, in clinical practice, biological responses and therapeutic effects induced by FLASH-RT may vary among patients, tumors, or treatment times, especially in combination with other treatment modalities (48). Thus, treatment scenarios must be thoroughly evaluated before FLASH-RT can be widely applied in clinic.

### 2.3 Preclinical and clinical studies of FLASH-RT

**Figure 1:**
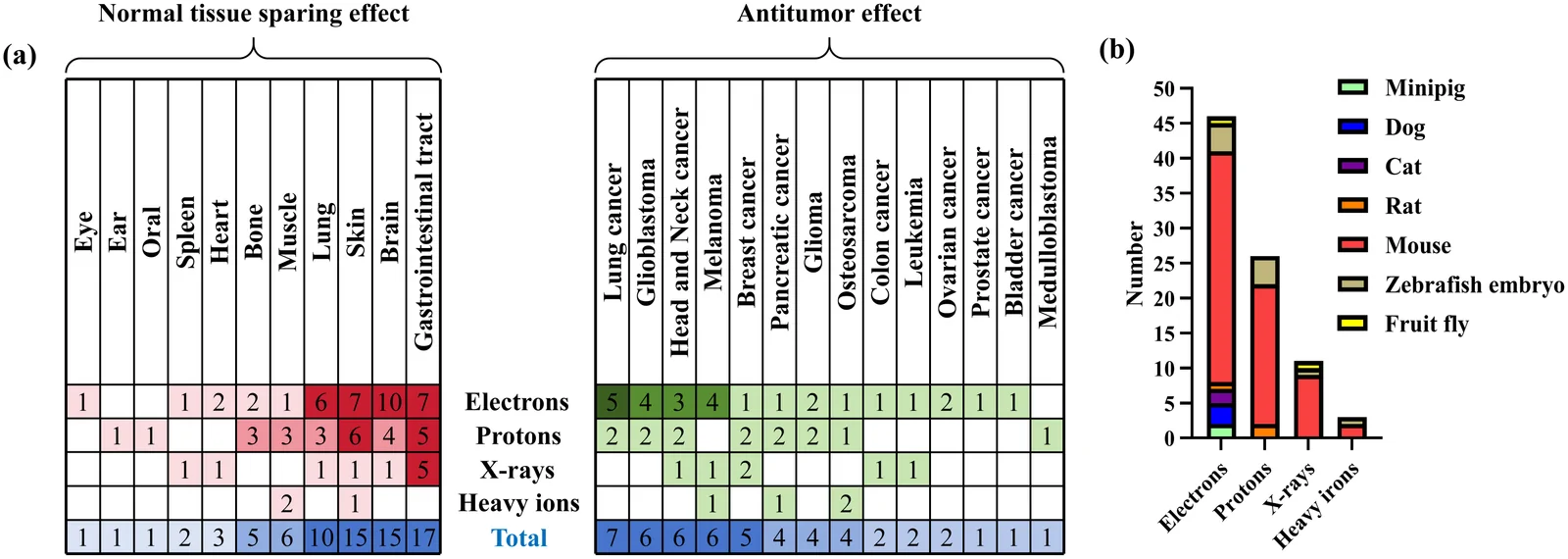
Preclinical *in vivo* studies of FLASH-RT (summarized studies published prior to May 2026). **(A)** Statistics of normal tissue sparing effects and antitumor effects across different normal organs and tumor types under various radiation beams (The color scale indicates study density). **(B)** Statistics of experimental models across various radiation beams.

Most preclinical studies have employed electron and proton radiation modalities, while X-rays and heavy ions are still under further development (**Figure 1**). Healthy tissue-sparing effects were primarily evaluated in the gastrointestinal tract, brain, skin, and lung. Additional organs, including bone, heart, spleen, and oral tissues, still require further study. Antitumor efficacy studies focused on lung cancer, glioblastomas, head and neck cancer, and so on. Experiments were mainly conducted using mouse model, while large animal, such as minipig, cat, and dog models also need further investigation.

Currently, clinical applications of both p-FLASH and e-FLASH have been reported. In one clinical case, p-FLASH produced encouraging results in a patient with cutaneous skin lymphoma (5), inducing a rapid, complete, and durable tumor response within 5 months, with only transient Grade 1 adverse events (AEs). Similarly, a non-randomized trial on the treatment of bone metastases with p-FLASH showed mild AEs, including four transient pain flares in 12 treated sites (49). Recently, in a phase I study for metastatic melanoma with dose escalation from 22 Gy to 28 Gy, FLASH-RT demonstrated no dose-limiting toxicities and no dose-dependent increase in acute skin toxicity, indicating favorable acute safety. Notably, the overall response rate reached 100% across all evaluable doses except 24 Gy, suggesting robust local antitumor activity (50). Ongoing clinical trials are exploring its use in bone metastases (NCT05524064, FAST-02), skin cancer (NCT05754875, Phase II) (ChiCTR2400080935) (ChiCTR2500106390), lung cancer (NL-OMON57588), and melanoma (NCT06549439, Phase I). However, current clinical evidence is confined to early-phase studies and small patient cohorts, and lacks randomized controlled trials to validate its superiority in curative-intent settings. Moreover, the median follow-up duration of these trials is less than 5 months, assessing acute toxicity only, while late-onset complications and chronic toxicities remain unevaluated.

## 3 Possible mechanisms of FLASH effect

**Figure 2:**
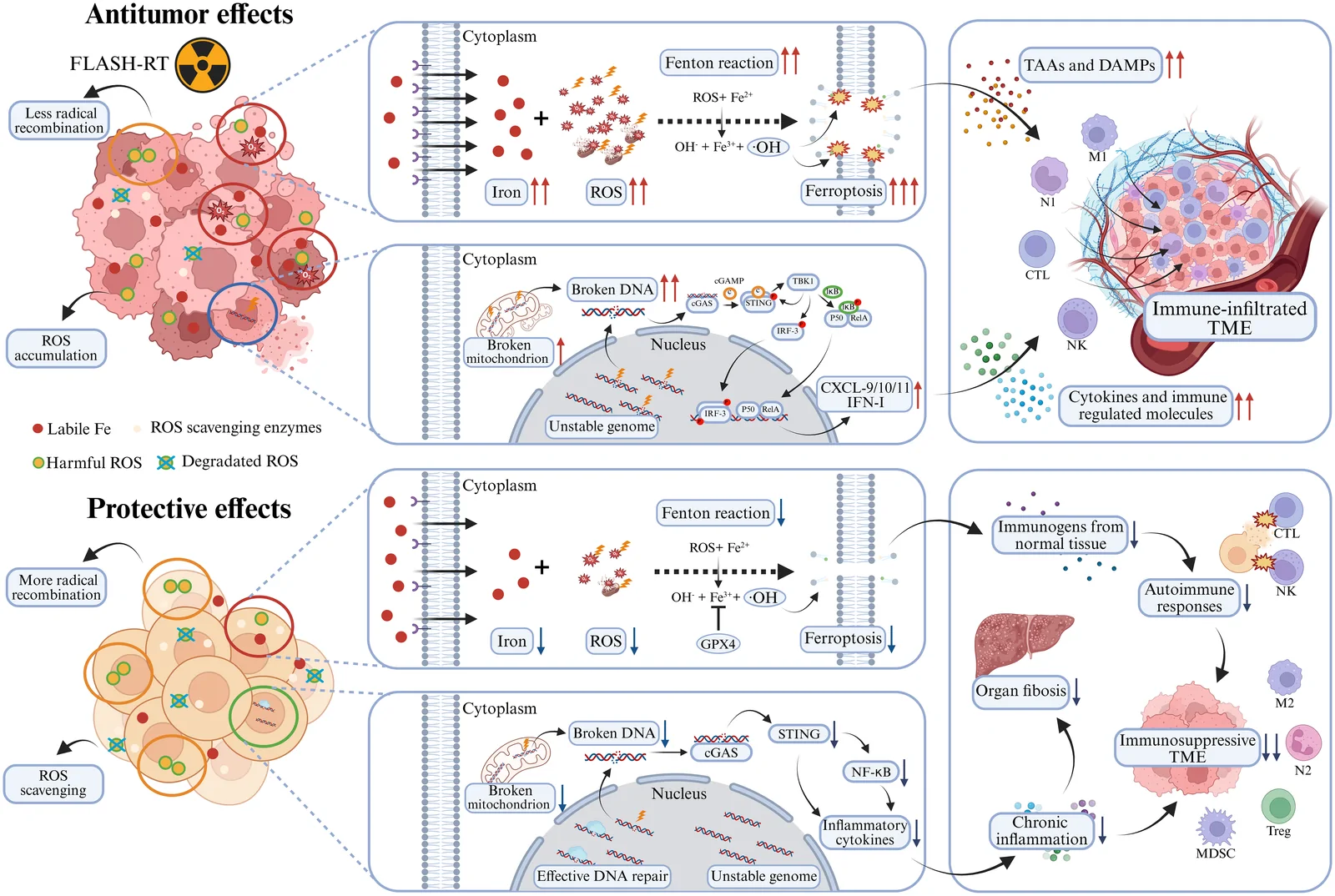
Possible mechanisms underlying the FLASH effect. Tumor tissue resides in an oxygen-poor and mitochondria-rich environment with a high level of Fe^2+^. It exhibits deficient radical recombination and ROS scavenging capacity. Under FLASH-RT, massive ROS released induce a relatively high level of the Fenton reaction, promoting tumor death by ferroptosis. Eventually, TAAs and DAMPs are released with the assistance of cytokines and other immune-regulated molecules to promote lymphocyte infiltration. Additionally, tumor cells have unstable genomes, inefficient DNA repair, and evaluated mitochondria content, making them more sensitive to instantaneous radiation exposure. This results in increasing accumulation of damaged DNA that activate the cGAS-STING pathway, leading to enhanced release of IFN-I and chemokines that boost the antitumor responses. In contrast, healthy tissue resides in an oxygen-rich environment with a low level of Fe^2+^ and high antioxidative capacity. Under FLASH-RT, the instant production of ROS in healthy tissue simultaneously increases the radical recombination, thereby reducing ROS toxicity. Moreover, the high ROS scavenging capacity of healthy tissue reduces the Fenton reaction and ferroptosis. Consequently, the release of healthy tissue derived immunogens is decreased, reducing autoimmune responses and the recruitment of immunosuppressive cells. Additionally, normal cells have a stable genome with effective DNA repair under instant radiation, which induce less activation of STING-mediated NF-κB signaling pathways, reducing chronic inflammation while preventing organ fibrosis. Created in BioRender, Dongliang, L. (2026) https://BioRender.com/4d0c764.

FLASH-RT exerts its antitumor effects through fundamental radiobiological mechanisms, including direct radiation-induced damage to genomic DNA, cellular membranes, and organelles, as well as indirect damage mediated by ROS (51). However, these mechanisms alone cannot fully account for the distinct toxicities observed between tumors and healthy tissues under FLASH-RT. In this section, we summarize current hypotheses related to the physical chemistry and biology of the FLASH effect. Well-established hypotheses have been partially validated but remain controversial, including oxygen consumption, DNA integrity and repair, and immune regulation, whereas emerging hypotheses remain in preliminary stages with limited evidence, encompassing mitochondria metabolism, iron metabolism, RNA integrity, vascular protection, and gut microbiota preservation (**Table 1**; **Figure 2**).

### 3.1 Oxygen consumption and ROS generation associated hypothesis

#### 3.1.1 Conventional oxygen depletion hypothesis

Oxygen depletion was the earliest hypothesis describing the FLASH effect, proposed in 1959 by Dewey and Boag. They suggested that ultra-high dose rates of electron radiation (600 rads/min) could deplete local oxygen, thereby reducing the generation of harmful ROS (52). This rapid dose delivery created conditions where the oxygen surrounding the irradiated area could not be replenished promptly. Later studies corroborated this idea, showing that ultra-high dose rates could induce transient hypoxia by significantly depleting local oxygen, which was thought to protect mammalian and tumor cells (53–56). These results indicated that oxygen and ROS concentration were crucial factors for the FLASH effect. However, this hypothesis was challenged by the possibility that tumor tissues might also be protected by transient hypoxia.

Recent evidence offers alternative explanations. One theory posits that healthy tissues, being naturally oxygen-rich compared to tumor tissues, benefit from the protective effects of transient hypoxia after FLASH-RT (6). Another theory highlights differences in hydrogen peroxide scavenging between healthy and tumor tissues (57). Healthy tissues have much higher catalase and superoxide dismutase (SOD) content and a lower oxidant load (58), enabling them to more easily and rapidly remove hydrogen peroxide than tumor tissues. Moreover, the radical interaction hypothesis suggests that when oxygen is rapidly consumed, ROS may be concurrently generated and react with one another, leading to rapid reorganization (59). Ultimately, FLASH-RT reduces ROS levels to prevent oxidative stress in healthy tissues (60–62).

Local oxygen concentrations also modulate the FLASH effect (63). *In vitro* studies show that in oxygen-rich healthy tissues (4–5% O_2_), ROS produced by FLASH-RT can transiently react with local oxygen, forming non-reactive species (64) and consuming local oxygen, thereby creating hypoxic conditions (dose >5–10 Gy to deplete the local oxygen) (59, 65). *In vivo* studies have further shown that oxygen-supplementation or vascular occlusion during FLASH-RT causes more brain and skin damage while blocking immune cell infiltration (66–68). Collectively, these findings suggest that sufficiently high doses and dose rates of FLASH-RT can rapidly deplete local oxygen in healthy tissues within a certain concentration range, thereby producing a protective hypoxic area.

However, the oxygen depletion hypothesis remains inconclusive due to several challenges. For instance, some *in vitro* studies have reported that well-oxygenated tumor tissues may also gain protective effect under FLASH-RT (69, 70). Conversely, an *in vivo* study indicated that tumor tissue radiosensitivity was unaffected by acute hypoxia during FLASH-RT (71). This discrepancy may arise from the limitations of *in vitro* monolayer cell cultures or simple 3D spheroid models, which fail to capture the spatial heterogeneity of oxygen distribution, vascular perfusion, tumor cell diversity, the complex tumor microenvironment, and variations in dose delivery that are inherent in *in vivo* experiments. Additionally, mitochondria-derived ROS and certain oxidases may be implicated in the FLASH effect (13). Some studies observed the FLASH effect in oxygen-rich organs, such as the lungs (72), while others suggested that FLASH-RT does not consume oxygen even at doses exceeding 30 Gy (73). Tissue oxygen concentration also varies depending on different anesthetic approaches, making it a non-negligible key factor in triggering the FLASH effect (74). In some cases, oxygen diffusion into hypoxic areas may negate the protective effects, adding further complexity to this hypothesis (75). Therefore, future investigations should pivot toward *in vivo* models that integrate real-time monitoring of intratumoral oxygen heterogeneity and vascular perfusion dynamics, utilize orthotopic tumor models with intact tumor microenvironments, and employ clinically relevant dose delivery systems to better recapitulate the spatial and physiological complexities governing the FLASH effect. Moreover, given the substantial inter-organ variability in oxygen tension and vascular perfusion across tissues such as the skin, intestine, lung, and brain, the dose and dose rate thresholds for oxygen depletion must be systematically characterized in an organ-specific manner.

#### 3.1.2 Mitochondria metabolism and iron metabolism hypothesis

Mitochondria generate oxygen-consuming energy through the tricarboxylic acid cycle (TCA) coupled with the electron transport chain and oxidative phosphorylation (OXPHOS) (76). When OXPHOS is disrupted by radiation, the electrons can leak extensively from mitochondria and react with oxygen, resulting in increased formation of mitochondrial-derived ROS, which may cause oxidative stress such as hypoxia, mutation induction, cell transformation, and cell death (77, 78). To adapt to the high proliferation rates under oxygen-poor or oxygen-rich conditions, tumor cells exhibit higher mitochondrial content than healthy tissues and display a marked dependence on glycolysis (the Warburg effect) (79), rendering them more sensitive to metabolic interference, particularly under FLASH-RT (71). Healthy tissues have lower mitochondrial content, regular energy metabolism, and a stronger antioxidant system, enabling them to better withstand ROS accumulation and oxidative toxicity. One hypothesis suggests that the ultra-rapid dose delivery of FLASH-RT can induce reverse electron transport. Under this condition, healthy tissue can enter metabolic hibernation to avoid damage from massive ROS generation, while tumor tissues fall into metabolic collapse and died from oxidative stress (80). Moreover, an *in vivo* study has proposed that FLASH-RT maintains mitochondria structure and function with reduced ROS generation in healthy cell which led by Dynamin-1-like protein (Drp-1), while inducing greater damage in tumor cells (62). A recent study further indicates that FLASH-RT maintains mitochondria structure and enhances antioxidant defenses through NRF2 upregulation (81). While direct evidence linking the FLASH effect to differential mitochondrial alterations in tumor versus healthy tissues remains lacking, accumulating date indicate that FLASH-RT spares muscle and brain tissue, which are considered to be mitochondria-rich, without compromising antitumor effects relative to CONV-RT (82–86).

Additionally, the differences in labile iron, ROS content, and pH between tumor and healthy tissues can explain the FLASH effect (57). Labile iron participates in cellular metabolism and proliferation and also reacts with ROS through the Fenton reaction (H_2_O_2_ + Fe^2+^ → ·OH + OH^-^ + Fe^3+^). Importantly, elevated Fenton reaction activity can induce the peroxidation of polyunsaturated fatty acid-containing phospholipids in cellular membranes, leading to the accumulation of lipid peroxides. This process can compromise membranes integrity, resulting in ferroptosis (87). Various tumor types exhibit significantly higher levels of labile Fe and transferrin receptors (88–90), rendering them more sensitive to Fenton reaction, especially following ROS generation induced by FLASH-RT. In contrast, healthy tissues can regulate and sequester endogenous labile Fe^2+^ and dependent and independent glutathione peroxidase 4 (GPX4) systems to inhibit ferroptosis (91, 92). This protective effect may be further enhanced when oxygen in healthy tissue is depleted by FLASH-RT. An *in vitro* study reported that FLASH-RT does not lead to lipid peroxidation in lipid micelles and liposomes (93). An *in vivo* study also suggested that FLASH-RT induces less lipid membrane damage in the intestine (94). Another study indicated that FLASH-RT induces substantial ferroptosis in tumor tissue but not in lung or intestine tissues, which is attributed to a high level of iron concentrations in tumor tissue (95). These findings support the hypothesis that FLASH-RT spares healthy tissue by reducing ferroptosis. Interestingly, FLASH-RT was found to produce a transient, highly acidic area (pH around 0.5–3.25 within 10^–8^ seconds) in irradiated tissue (96), which is consistent with the finding that the Fenton reaction exerts maximal efficiency at pH 2.5-3.5 (97). These findings suggest that this transient, strongly acidic area represents a potential factor influencing the FLASH effect.

### 3.2 DNA integrity and damage signaling-associated hypotheses

#### 3.2.1 cGAS-STING pathway: a DNA sensor activating immune responses

Compared with CONV-RT, FLASH-RT induces fewer γH2AX and 53BP1 foci (72, 98, 99), indicating that FLASH-RT causes less double-stranded DNA (dsDNA) damage. Moreover, dsDNA serves as a key activator of the cyclic GMP-AMP synthase-stimulator of interferon genes (cGAS-STING) pathway, which involves interferon regulatory factor 3 (IRF3) and canonical/non-canonical nuclear factor kappa-B (NF-κB) signaling. Initially, dsDNA is recognized by the cytoplasmic nucleic acid sensor cGAS, which palmitoylates STING to activate IRF3. Together with p50-RelA, they govern the activation of downstream immunomodulatory signaling (100, 101). Instantaneous delivery of FLASH-RT protects DNA integrity in healthy tissues, resulting in less dsDNA breakage (10), further reducing cGAS-STING activation and preventing detrimental immune reactions in healthy tissues. In contrast, tumor tissues have more unstable genomic DNA and an inefficient DNA repair system (102), leading to increased DNA damage and cGAS-STING pathway activation compared to healthy tissue after FLASH-RT. Moreover, FLASH-RT reduces single-stranded DNA (ssDNA) breakage, but dsDNA breakage remains unchanged (103). ssDNA breakage is much easier to repair via base excision repair (BER) than dsDNA breakage, further enabling healthy tissues to repair damage more effectively (104). However, unresolved issues remain. For example, the role of three prime repair exonuclease 1 (TREX1) under FLASH-RT remains unclear, as it can degrade broken ssDNA and dsDNA in the cytoplasm (105), leading to the failure of cGAS-STING pathway activation, especially at doses above 8 Gy (106). In addition to nuclear and cytoplasmic DNA damage, the mitochondrial genome (mtDNA) may serve as another source of cGAS-STING pathway activation (107). Under irradiation, mtDNA can be released by damaged mitochondria, especially in tumor tissues with elevated mitochondrial content and inefficient mtDNA repair (108), rendering them more sensitive to irradiation. FLASH-RT could reduce mitochondrial damage in healthy tissue, indicating that FLASH-RT can protect more mtDNA from radiation-induced injury (62). However, the specific mechanisms remain unclear.

#### 3.2.2 RLR-MAVS pathway: an RNA sensor activating immune responses

Similar to cGAS, retinoic acid-inducible gene I (RIG-I)-like receptors (RLRs), a major type of PAMPs to recognize RNA, containing RIG-I, melanoma differentiation associated gene 5 (MDA5), and laboratory of genetics and physiology 2 (LGP2) participated in the recognition of abnormal RNA to activate the immune system (109). RIG-I and MDA5, the primary RLR members, are activated by ssRNA or dsRNA. This activation recruits mitochondrial antiviral signaling protein (MAVS), which promotes IRFs (IRF3, IRF7) and NF-κB activation via TANK-binding kinase 1 (TBK1) and inhibitor of kappa B kinase (IKK) signaling (110). This process results in the release of IFN-I and other pro-inflammatory cytokines, initiating immune responses (111). RNA damage and ROS induced by radiation can activate the RLR-MAVS pathway via MAVS oligomerization (112, 113), actively enhancing DC-mediated T cell responses (114). MDA5 can also be activated by dsRNA generated by endogenous retroviruses (ERV) (115). Moreover, following 8 Gy radiation, RIG-1 was significantly upregulated, indicating that radiation can trigger antitumor responses via the RLR-MAVS pathway (116). However, the roles of the RLR-MAVS pathway in CONV-RT and FLASH-RT remain to be fully elucidated.

### 3.3 Immunological capacity of FLASH-RT

#### 3.3.1 FLASH-RT spares the circulating immune cells and reduces irradiated blood volume

The ultra-rapid dose delivery of FLASH-RT minimizes radiation exposure to the immune system and the volume of irradiated blood, thereby preserving the priming capacity of antitumor immunity while reducing immunosuppression. A simulation study showed that the mortality rate of circulating immune cells under FLASH-RT (5–10%) was significantly lower than that under CONV-RT (90–100%) (117). Another study using a dosimetric blood flow model for brain cancer proposed that FLASH-RT with a single dose, irradiated 1.5% of peripheral blood, while hypofractionated FLASH-RT irradiated 7.3% of the same tissue, reducing the depletion rate of circulating blood and lymphocytes by 69.2% (118). In a HEDOS simulation model, FLASH-RT demonstrated significant potential in minimizing lymphocyte depletion, thereby maintaining the absolute lymphocyte counts and facilitated lymphocyte recovery (119). By contrast, 42.4% of peripheral blood was exposed to irradiation under hypofractionated CONV-RT. An *in vivo* study indicated that compared to CONV-RT, FLASH-RT is more sensitive to T-cell acute lymphoblastic leukemia (T-ALL) than to immune cells and other blood cells, while inducing less damage to blood stem cells (120). Furthermore, FLASH-RT induced higher levels of apoptosis in malignant tumors than in healthy lymphocytes, and induced lower levels of apoptosis in immune cells compared to CONV-RT (121, 122). FLASH-RT also spared activated T cells in tumor-draining lymph nodes and within tumors (123). These results indicate that the very short delivery time of FLASH-RT protected circulating or infiltrated immune cells and decreased the irradiated volume of blood at both single and hypofractionated doses, which reduced the release of pro-inflammatory signaling and depleted pro-survival signaling (80). Although irradiated blood can enter whole-body circulation, its volume is still too small to induce sufficiently strong toxic responses. For CONV-RT, a massive volume of blood exposed to radiation induces abundant pro-inflammatory signaling, and pro-survival signaling is depleted, which can spread through vessels to induce toxic responses throughout the body. Moreover, excessive recruitment of inflammatory immune cells and cytokines contributes to the establishment of fibrosis in healthy tissues (124). More importantly, these inflammatory components trigger antitumor immune cells to transform into pro-tumor phenotypes, supporting tumor escape from the immune system and leading to the formation of an immunosuppressive TME.

#### 3.3.2 FLASH-RT creates a moderate immune-infiltrated TME

**Figure 3:**
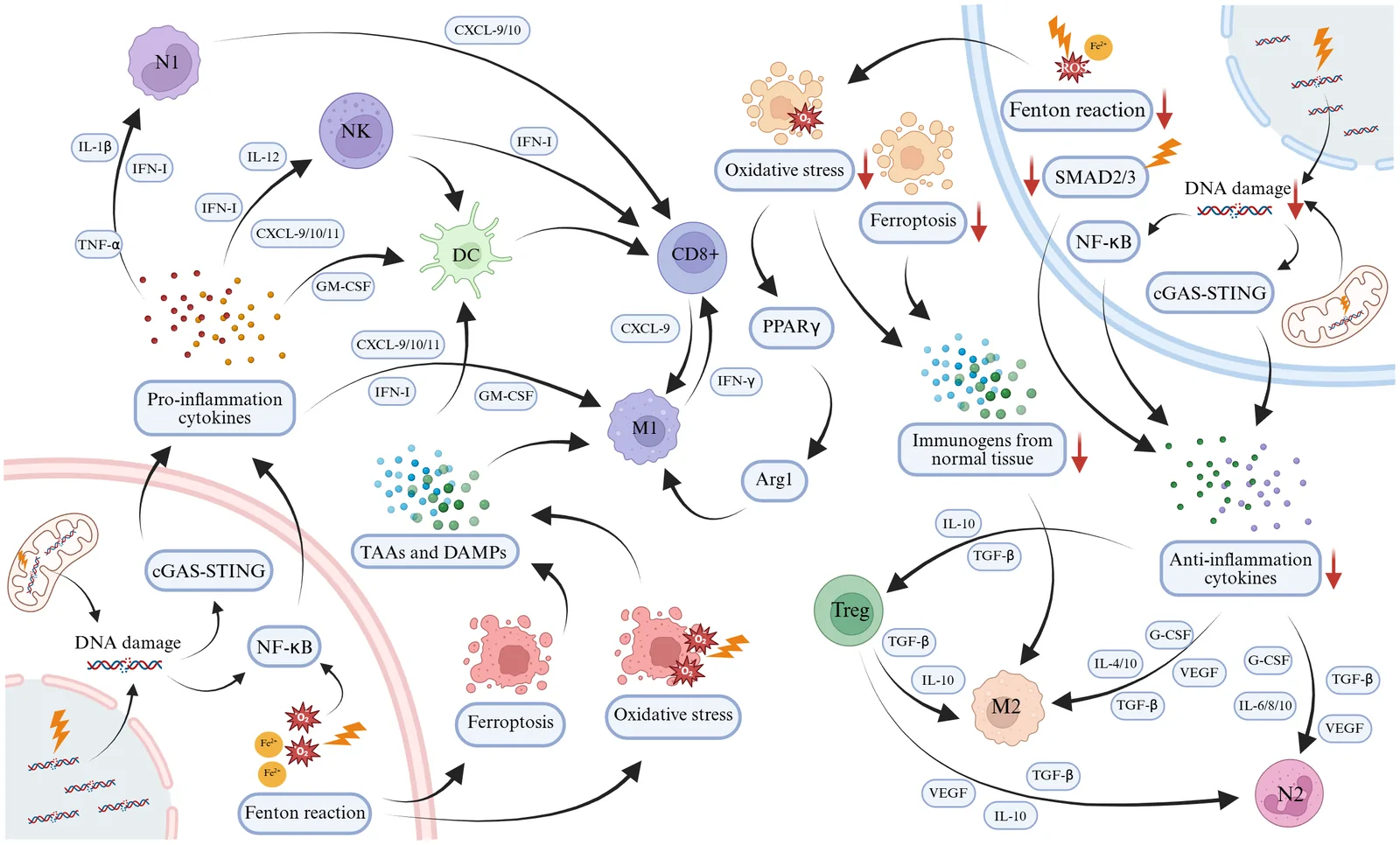
Crosstalk between immune cells under FLASH-RT. FLASH-RT promotes CD8^+^ T cells, NK cells, M1 cells, and N1 cells infiltration by enhancing the release of pro-inflammation cytokines, including IFN-I, CXCL-9/10/11, IL-12, GM-CSF, TNF-α), as well as DAMPs (HMGB1, ATP, CRT) and TAAs. M1 and CD8^+^ T cells reciprocally promote each other via IFN-γ and CXCL9 secretion. Meanwhile, FLASH-RT protects normal tissue by reducing ROS induction, then attenuating M2 polarization through PPARγ-Arg1 pathway. Moreover, owing to its normal tissue protective effect, FLASH-RT decreases the release of immunosuppressive factors, such as IL-10, VEGF, G-CSF, and TGF-β, which are critical for M2, N2, and Treg cells polarization and infiltration. Created in BioRender, Dongliang, L. (2026) https://BioRender.com/61ggse7.

Under FLASH-RT, a broad spectrum of immune cells and molecular regulators undergo substantial alterations (**Figure 3**). Similar to CONV-RT, FLASH-RT kills tumors through direct DNA damage and ROS-related indirect cytotoxicity, promoting the release of tumor immunogens, and then enhancing the recruitment and infiltration of antitumor immune cells (13, 28, 125–127). However, owing to its protective effects, FLASH-RT induces a distinct TME that differs from that induced by CONV-RT. On the one hand, FLASH-RT maintains its antitumor capacity, which reprograms the immunosuppressive TME into an immune-infiltrated phenotype by promoting tumor-destroying CD8^+^ T cells and tumor-killing macrophages (M1) infiltration (12, 13, 128, 129). Mechanistically, decreased ROS of FLASH-RT promotes activation of M1 through the ROS-PPARγ-Arg1 pathway (13), and then M1 facilitates the antitumor capacity of CD8^+^ T cells by releasing IFN-γ. CD8^+^ T cells reversely promote M1 polarization by releasing CXCL9 (12). Moreover, FLASH-RT downregulates the Chk1-STAT3 pathway to accelerate the recovery of CD8^+^ T cells, CD3^+^ T cells, NK cells, and B cells (122). On the other hand, FLASH-RT reduces radiation-induced toxicity to immune organs, lymphocytes, and vasculature, thereby decreasing the recruitment of immunosuppressive cells including regulatory T cells (Tregs) and tumor-promoting macrophages (M2), and dampening inflammatory pathways such as NF-κB and cGAS-STING (12, 13, 58, 129, 130). This enables FLASH-RT to maintain durable and mild antitumor responses, thereby reducing the rapid re-establishment of an immunosuppressive TME, chronic inflammation, and organ fibrosis.

Inflammatory cytokines such as IFN-I, TGF-β, granulocyte-colony stimulating factor (G-CSF)/granulocyte-macrophage colony stimulating factor (GM-CSF), TNF-α, IL-6, IL-1β are key driving factors. Among them, TGF-β is a crucial cytokine that can be markedly upregulated after radiation, and is directly correlated with the radiation dose (131). Under CONV-RT, TGF-β accumulates massively, promoting fibrosis in the lung, liver, kidney, heart, intestine, and other organs via the TGF-β-SMAD2/3 pathway (132–134). Moreover, TGF-β promotes tumors stemness, growth, invasion, and metastasis and recruits immunosuppressive cells, thereby facilitating the formation of an immunosuppressive TME (135, 136). However, TGF-β expression under FLASH-RT is significantly decreased compared to that under CONV-RT (total dose >10 Gy) (4, 85, 137, 138). This change positively enhances antitumor immune responses and reduces immunosuppression and organ fibrosis. Moreover, under FLASH-RT, TGF-β is also involved in suppressing excessive inflammation, facilitating tissue repair by epithelial–mesenchymal transition (EMT) (139). Nevertheless, some researchers proposed a contrasting hypothesis that decreased TGF-β levels induced by FLASH-RT might impair the radioprotective effects on T cells (140, 141). Regarding IFN-I, FLASH-RT promotes its release from CD8^+^ T cells through activating the cGAS-STING pathway, which further induces M1 activation. These activated M1 secrete IFN-γ in turn, promoting the activation of CD8^+^ T cells (12). Notably, FLASH-RT induces relatively weak IFN-I responses in the early stage that gradually gain strength by the late stage (a transient pattern), which helps to protect healthy tissue by reducing chronic inflammation, while maintaining the activation of long-term memory T cells (29, 142). Inflammatory cytokines such as CXCL-1 and G-CSF decreased after FLASH-RT (138), which may reduce the infiltration of tumor-promoting M2 and protumorigenic neutrophils (N2) (143, 144), and mitigate tissue injury and chronic inflammation (145). Additionally, compared with CONV-RT, IL-6 and IL-1β levels were significantly lower under FLASH-RT, thereby reducing the chronic inflammation to protect healthy tissue (146). Regarding immune-regulated molecules, FLASH-RT exerts considerable healthy tissue-sparing effects compared to CONV-RT, which decreases the release of healthy tissue immunogens, further reducing the recruitment of immunosuppressive components and preventing autoimmune damage to normal tissues. Levels of damage-associated molecular patterns (DAMPs), including high mobility group box protein 1 (HMGB1) and related molecules, were decreased in healthy tissues under FLASH-RT, thereby attenuating autoimmune responses (83). For tumor cells, FLASH-RT maintains its antitumor capacity by inducing tumor cells to release tumor-associated antigens (TAAs) and DAMPs, which stimulate immune system activation (147). Regarding surface ligands on tumor or immune cells, major histocompatibility complex-II (MHC-II) and MHC-I are upregulated under FLASH-RT, which promotes the recognition and presentation of tumor antigens (127, 148). Compared to CONV-RT, upregulation of immune checkpoint ligands, including PD-L1, was attenuated in tumor cells, whereas these ligands remained upregulated in immune cells under FLASH-RT (129, 148). Concurrently, PD-1 and mucin domain-containing protein 3 (TIM-3) expression remained largely unchanged in immune cells (13), limiting the recruitment and infiltration of Tregs, M2, and other immunosuppressive cells (149). FLASH-RT also positively activates the immune system, even in a model of severe immunodeficiency, compared to CONV-RT (28). An enhanced abscopal effect has also been observed under FLASH-RT (12, 82).

**Figure 4:**
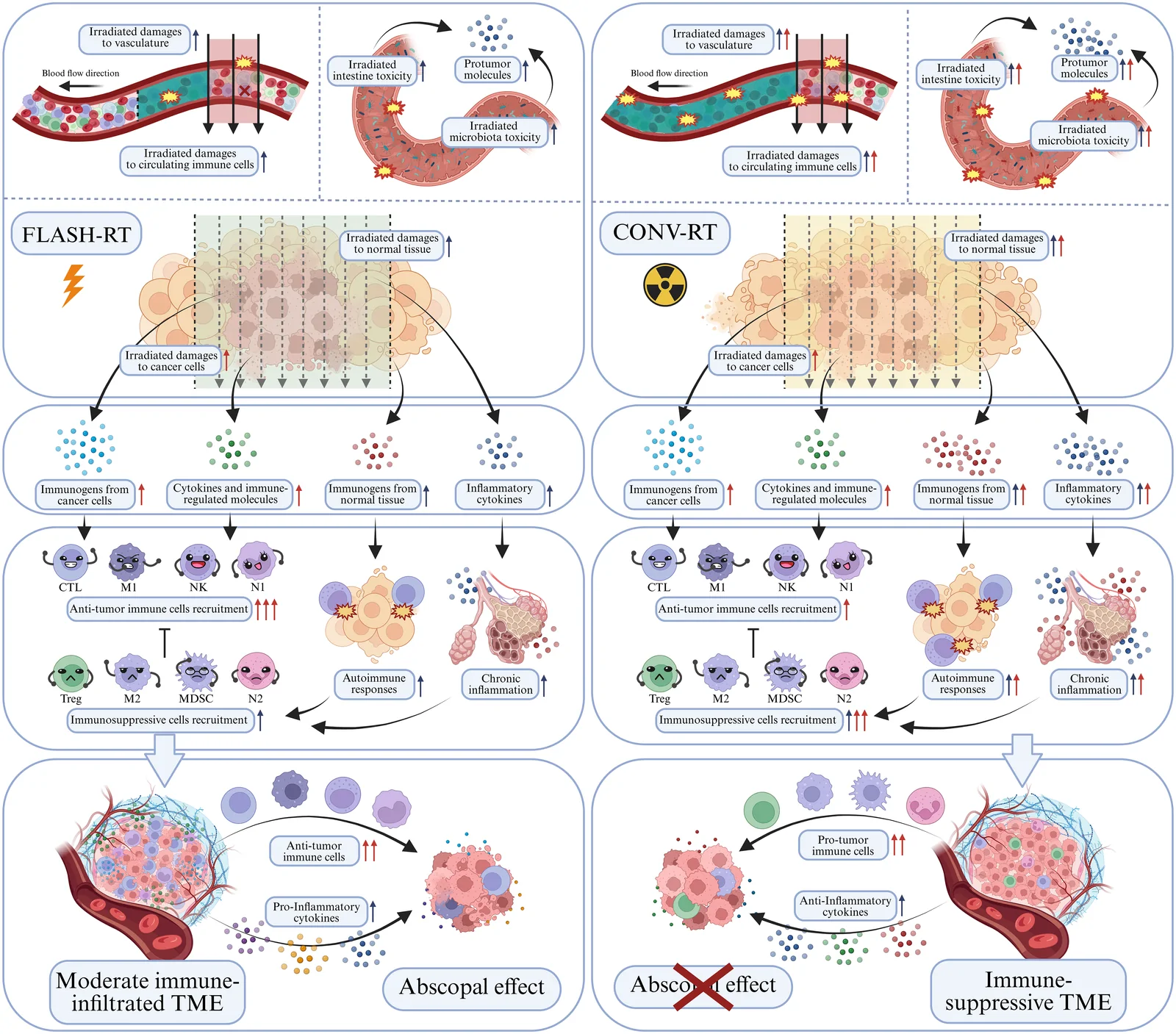
Comparison of the ability to activate TME between FLASH-RT and CONV-RT. FLASH-RT reduces radiation-induced toxicity to healthy tissue, including circulating and infiltrated immune cells, vasculature, and intestine. This inhibits the release of inflammatory cytokines that alleviate acute and chronic inflammation. Moreover, FLASH-RT reduces the release of immunogens from healthy tissue, which prevents autoimmune responses. These protective effects of FLASH-RT lead to less recruitment of immunosuppressive cells. Simultaneously, FLASH-RT damages tumor cells and promotes the release of TAAs and antitumor immunogens, facilitating the recruitment of antitumor immune cells. Consequently, the immunosuppressive TME converts into a moderately immune-infiltrated one, facilitating maintenance of durable antitumor immune responses, tissue repair, stimulation of memory T cells, and enhancement of abscopal effect. Created in BioRender. Dongliang, L. (2026) https://BioRender.com/ui21kuj.

Collectively, FLASH-RT engenders an immune-infiltrated TME fundamentally distinct from that shaped by CONV-RT (**Figure 4**). FLASH-RT downregulates pro-inflammatory signaling by mitigating the radiation-induced toxicity, thereby attenuating systemic inflammation. This modulates the speed at which lymphocytes enter the TME, eliciting moderate yet robust antitumor responses. Moreover, immunosuppressive factors do not accumulate rapidly, eventually restraining the recruitment of immunosuppressive cells and the development of chronic inflammation. Most importantly, this durable antitumor immune response promotes healthy tissue repair, fosters T cell memory, and enhances the abscopal effect. Consequently, FLASH-RT delays the rapid re-establishment of an immunosuppressive TME and limits late-stage organ fibrosis. In contrast, CONV-RT induces relatively high radiation-induced toxicity, triggering a massive pro-inflammatory wave that strongly and rapidly activates the immune system and lymphocytic infiltration into the TME. These “fierce” antitumor immune responses are accompanied by rapid and persistent accumulation of anti-inflammatory signaling, which fosters the development of an immunosuppressive TME and organ fibrosis. Moreover, the more extensive healthy tissue damages under CONV-RT results in increased self-tissue-derived immunogenic substances released, heightening the risk of autoimmunity and redundant immune responses against the host.

Notably, FLASH-RT may attenuate the infiltration of tumor-associated fibroblasts (TAFs), tumor-associated neutrophiles (TANs) and MDSCs. However, the underlying mechanisms remain largely unexplored. Tumor cells and immunosuppressive cells release TGF-β, IL-1β, platelet-derived growth factors (PDGF) and fibroblast growth factor 2 (FGF2), which possess a remarkable capacity to transform normal fibroblasts into TAFs (150). In turn, TAFs foster tumor invasion and recruit immunosuppressive cells by releasing TGF-β, IL-4, IL-6, IL-10 (151). Regarding TANs, TGF-β, IL-8, IL-10 and PGE-2 contribute to recruit N2 (152). Then, N2 can upregulate PD-L1 to inhibit NK, DCs and T cells, and promote tumor progression via VEGF and CXCR4 release (153). In concerning to MDSCs, a complex array of cytokines such as GM-CSF, TGF-β, IFN-γ, VEGF, IL-4, IL-6 and IL-10 participates in their recruitment (154). MDSCs promote immunosuppression by releasing TGF-β, IL-6, IL-10, arginase, iNOS, COX2, rendering strong inhibition to CD8+ T cells and NK cells, promoting the activation of M2, and favoring the naïve CD4+ T cells conversed to Tregs (155, 156). When normal tissues are exposed to radiation toxicity, these immunosuppressive effects mediated by TAFs, N2, MDSCs may be further exacerbated due to the massive release of inflammatory factors (157, 158). According to current studies, FLASH-RT reduces these key driving immunosuppressive factors, including TGF-β, IL-4, IL-6, IL-10, and IL-1β, then potentially attenuating the infiltration of TAFs, N2, and MDSCs. However, further investigation is needed to elucidate the infiltration of these cells and the underlying driving mechanisms.

Nevertheless, some studies have yielded inconsistent results, demonstrating that FLASH-RT triggers similar antitumor immune responses relative to CONV-RT, with comparable immune-cell populations (CD8+ T cells, NK cells and Tregs) and cytokine profiles (TGF-β, IL-2, IL-6, IL-1β, GM-CSF, and TNF-α) observed in the TME under both radiotherapy regimens (28, 29, 127, 142, 147, 159). Such divergent outcomes reflect the interplay of multiple factors, including distinct dosimetric parameters (dose- and dose rate thresholds governing immune responses and radiation-induced fibrosis), temporal windows (acute tissue repair versus chronic tissue remodeling), and experimental models (different organ types and animal species). Future investigations are still needed to decipher the timeline-dependent patterns, organ-specific radio-susceptibilities, and dose-response relationships underlying these molecular alterations.

#### 3.3.3 Other pathways that affect immune responses under FLASH-RT

The FLASH effect also occurs in the vasculature. FLASH-RT preserves its capacity to damage tumor vasculature, the blood vessels of which are disorganized, immature, and permeable due to the high levels of pro-angiogenic factors released by tumor cells (160). CONV-RT also possess the same ability to damage tumor vasculature. However, it also induces toxicity to healthy blood vessels, especially microvasculature in a dose-dependent manner (161), leading to acute apoptosis particularly for endothelial cells (162, 163), accompanied by overexpression of VEGF, angiopoietins, TGF-β, and other pro-angiogenic factors that promote tumor growth and invasion (164). Under FLASH-RT, the volume of blood vessels is maintained (61), which reduces acute apoptosis of blood vessels and protects the integrity of the microvasculature in the brain. Moreover, FLASH-RT reduces rapid activation of myosin light chain (MLC), which is involved in cellular contraction (126), preventing vascular collapse. The sparing effects of FLASH-RT were also observed in the endothelium, as the expression of CD31, which regulates vascular integrity and immune cell trafficking, was significantly higher under FLASH-RT than under CONV-RT (83). Additionally, FLASH-RT preserved microvasculature by protecting Aquaporin 4 (165).

Notably, FLASH-RT exerts a smaller impact on the gut microbiota. Under CONV-RT, various intestinal microbiota species experienced marked changes that could influence the digestive, genital, hematopoietic, and respiratory systems, promoting the progression of tumors, such as colorectal, gastric, hepatocellular, and cervical cancers (166). Radiation-induced alterations in the ratio of harmful bacteria to beneficial bacteria may be a key factor contributing to regulate immune responses (167). These immune responses were significantly driven by inflammatory signaling pathways and cytokines, including the NF-κB pathway, IL-17, IL-23, and TGF-β (168–170), microbiota metabolites, such as short-chain fatty acids (171, 172), PAMPs (173), and various immune-regulated molecules (174). More seriously, CONV-RT induces mucositis, diarrhea, enteritis, and other severe gastrointestinal adverse effects, caused by radiation-induced intestinal epithelial injury, intestinal microvascular damage, and immune responses, which may strengthen the connection between the host and gut bacteria (175). Eventually, gut microbiota can become dysfunctional, boosting the growth, invasion, and metastasis of tumors by altering host genes, inducing an inflammatory TME, and releasing metabolic byproducts (176). However, compared to CONV-RT, FLASH-RT causes fewer alterations in the gut microbiota, less damage to the intestinal crypt cells, and less fibrosis (18, 177, 178). Even in a whole-brain irradiation model, FLASH-RT upregulated probiotics such as *Lachnospiraceae*, which protect against radiation (179). Hence, the lower radiation-induced toxicity to the gut microbiota and healthy gastrointestinal tissue could result in better therapeutic efficacy than that achieved by promoting tumors through the action of gut bacteria.

## 4 Preclinical studies and potential of combining FLASH-RT with immunotherapy

### 4.1 Evidence supporting FLASH-RT combined with immunotherapy

Recent studies have demonstrated the application of FLASH-RT in combination with immunotherapies such as anti-PD-1, anti-PD-L1, and anti-CTLA-4 antibodies, as well as CAR-T, STING agonist MSA-2, and the TLR-7 agonist imiquimod (**Table 2**). Preliminary findings suggest that FLASH-RT combined with immunotherapy is feasible and safe.

**Table 2:** Preclinical studies combining FLASH-RT with immunotherapy.

Model	FLASH-RT (dose × fraction)	Immunotherapy (administration timing)	Main findings	Mechanism	Ref.
Colon cancer (Mice)	X-rays (110–120 Gy/s, 13 Gy × 1f, 5 Gy × 5f)	Anti-PD-L1 (knock out)	FLASH-RT protected the immune system (T cells), structure and function of intestine	Decreased activation of cGAS-STING in healthy tissue and pyroptosis of CTL reduces inflammation	(10)
Drug-resistant ovarian cancer (Mice)	Electrons (210 Gy/s, 14 Gy × 1f)	Anti-PD-1 (Day +7, +10, +13)	FLASH-RT reduced radiation-induced toxicity to intestine, enhanced antitumor efficacy and increased toxicity when combined with anti-PD-1	Increased infiltration of CD8^+^ T cells and decreased Tregs	(11)
Lung cancer (Mice)	Electrons (Hypofractionated 8 Gy × 3f)	Anti-CTLA-4 (Day -1, 0, + 2)	Anti-CTLA-4 with FLASH-RT or CONV-RT had the same local or systemic immune responses	–	(28)
Medulloblastoma (Mice)	Protons (80–135 Gy/s, 10 Gy × 1f)	GD2 CAR-T (Day +5)	FLASH-RT promoted activation of M1 and CD8+ T cells, while enhancing the infiltration and antitumor efficacy of CAR-T	FLASH-RT abrogated lipid oxidase expression and oxidized low-density lipid generation to reduce PPAPγ and arginase 1 expression	(13)
Melanoma (Mice)	Protons (95–103 Gy/s, 10 Gy × 1f)	Imiquimod (IMQ, TLR-7 agonists, Day -1)	FLASH-RT promoted the release of IMQ, enhancing antitumor efficacy	Increased release of G-CSF, IL-9, IP-10, MCP-1, MCP-2 and VEGF	(180)
Breast cancer (Mice)	X-rays (>40 Gy/s, 10 Gy × 1f)	MSA-2 (STING agonists, Day -1)	FLASH-RT promoted the release of MSA-2, enhancing antitumor immune responses and long-term memory ability	Increased recruitment of DC and CTL, and increased release of IFN-γ, IL-6	(181)

In an ovarian cancer model (11), FLASH-RT showed comparable antitumor immune responses and abscopal effect compared to CONV-RT. However, FLASH-RT retained more regenerating crypts, protected the function and structure of the intestine. Moreover, compared with CONV-RT, FLASH-RT increased CD8^+^ T cells infiltration, while decreasing Tregs accumulation. Similarly, in anti-PD-1 resistant models, only FLASH-RT combined with anti-PD-1 still reduced the infiltration of Tregs and M2, while increasing CD8^+^ T cells population. The combination also significantly reduced tumor weight and ascites volume, indicating both superior efficacy and safety. In anti-PD-1 sensitive models, FLASH-RT enhanced CD8^+^ T cell infiltration without affecting body weight, stool production, or complete blood counts, suggesting no additional toxicity.

Another study using a whole abdominal model in PD-L1 knockout mice reported that FLASH-RT protected the structure and function of the intestine compared to CONV-RT (10). Over 6 months, FLASH-RT reduced chronic intestinal inflammation and fibrosis. Levels of cytotoxic T lymphocytes (CTLs) in intestinal crypts were significantly lower than those with CONV-RT. Mechanistically, FLASH-RT triggered less CTL death via GSDME-mediated pyroptosis, which further lowered the expression of chemokines such as CCL5, CXCL9, CXCL10, and CXCL11. These reductions consequently promoted CD8^+^ T cell infiltration, resulting in diminished inflammation and autoimmunity. Moreover, FLASH-RT reduced DNA damage in healthy tissue, thereby downregulating activation of the cGAS-STING pathway, leading to an additional decrease in chronic inflammation.

Compared with CONV-RT, FLASH-RT significantly promoted the infiltration of CD3^+^, CD8^+^ T cells, as well as M1, and concurrently elevated the ratio of CD8^+^/CD4^+^ T cells and M1/M2 (13). This enables FLASH-RT to create a TME distinct from that generated by CONV-RT. Mechanistically, FLASH-RT reduced the level of ROS, thereby decreasing Arg expression through the ROS-oxLDL-PPARγ-Arg pathway, resulting in enhanced recruitment of M1 and CTLs. Moreover, markers of T cell exhaustion, including PD-1 and TIM-3 were downregulated. These changes enhanced the activation and infiltration of GD2 CAR-T cells and sensitized medulloblastoma to GD2 CAR-T cell therapy. Eventually, FLASH-RT combined with GD2 CAR-T prolonged the median survival of mice to 27.5 days, more than doubling the median survival observed with CONV-RT plus GD2 CAR-T (11 days).

Some studies are also testing multifunctional materials that carry immune-regulated drugs to precisely control the antitumor immune responses. On the one hand, FLASH-RT has been applied as a tool to promote the release and absorption of immune-regulated drugs, including TLR-7 agonists (180) and STING agonists (181). On the other hand, these immune-regulated drugs promoted the infiltration of antitumor immune cells such as DCs and CTLs, without enhancing toxicity to healthy tissues. Meanwhile, these drugs enhanced the antitumor efficacy of FLASH-RT.

However, in a study combing hypofractionated FLASH-RT and anti-CTLA-4 in a lung cancer model (28), local and systemic immune benefits were not observed, with results nearly identical to those of CONV-RT combined with anti-CTLA. These results contrast with those of a previous study that used the same tumor model and dose (8 Gy×3 fractions) of hypofractionated CONV-RT with anti-CTLA-4 (182). Hence, whether FLASH-RT combined with anti-CTLA-4 can promote antitumor immune responses requires further investigation.

In summary, when combined with immunotherapy, FLASH-RT does not exacerbate toxicity and enhances antitumor responses even in ICI-resistant models. These findings support the hypothesis that the moderately immune-infiltrated TME induced by FLASH-RT provides a potential basis for combination with immunotherapy. Nevertheless, further studies are needed to investigate the optimal regimens, sequencing, and long-term safety.

### 4.2 CONV-RT acts as a double-edged sword in TME

RT positively participates in antitumor immune responses and the TME remodeling process (183, 184). Under CONV-RT, these components, including immune cells, cytokines, and surface molecules, undergo significant alterations, determining the eventual efficacy of immune responses, which can be pro-tumor or antitumor (185). In early stages, CONV-RT exerts significant antitumor immune responses by promoting the release of TAAs, DAMPs, and cytokines; boosting the expression of MHC molecules and co-stimulating ligands (186, 187); enhancing the antigen presentation capacity of APCs; and promoting lymphocyte infiltration and their antitumor capacity (188). During the initial RT period, antitumor immune responses are most active, which can reshape a “cold” TME into a “hot” one and trigger the occurrence of the abscopal effect (189). In contrast, CONV-RT simultaneously induces serious irradiation toxicity in healthy tissues, the immune system, and the vasculature. To avoid healthy tissue injury, anti-inflammatory cytokines like TGF-β, IL-4, IL-6, and IL-10 are upregulated to recruit immunosuppressive cells such as Tregs, MDSCs, M2, N2 to inhibit harmful immune responses (190, 191). Conversely, this facilitates tumor escape from the immune system. More seriously, to avoid recognition by the immune system, various types of tumor cells can also express remarkably high levels of immune checkpoints like PD-1/L1, CTLA-4, and TIM-3, and release anti-inflammatory molecules. When these immunosuppressive components accumulate to surpass the antitumor immune responses, an immunosuppressive TME is formed, resulting in the promotion of tumor growth, invasion, and loss of the abscopal effect. Therefore, minimizing healthy tissue toxicity while targeting more tumor tissues to preserve antitumor immunity is crucial. Equally important are strategies to delay the formation of immunosuppressive TME formation at early stages of disease progression.

### 4.3 Clinical bottlenecks in CONV-RT combined with immunotherapy

Immunotherapy activates the immune system to attack tumors, encompassing ICIs, ACTs, and cancer vaccines (192). To date, ICIs such as anti-PD-1/PD-L1, anti-CTLA-4 and other immune-regulated drugs have been widely studied and applied in the clinic (193). However, treatment with ICIs alone is only effective when the tumor burden is small in the early stages of the disease (194), only a fraction of patients obtain benefits, with an actual response rate of 10–30%, and many even suffer from drug-related adverse effects (195). Regarding ACTs, chimeric antigen receptor T cells (CAR-T) and CAR-NK therapy have achieved breakthroughs in the treatment of drug-resistant hematological tumors (196). However, they are inefficient for the treatment of solid tumors owing to the escape of tumor antigens, insufficient infiltration, and immunosuppression in the TME (197). When combined with immunotherapy, RT provides a non-pharmacological, low-toxicity, and highly effective approach to increase the response rate of ICIs by activating the immune system (198, 199). Conversely, ICIs offer a suitable method to control the progress of immunosuppression caused by radiation, achieving more active antitumor immune responses in late treatment. For ACTs, RT triggers the infiltration of lymphocytes into solid tumors to boost the ACT response rate and can also be a bridging treatment approach prior to ACTs to reduce the tumor load. In contrast, ACTs are more precise in attacking targeted tumors, especially metastatic tumors, and can eradicate radio-resistant or drug-resistant CSCs (200, 201). To date, clinical studies combining RT with ACTs have been rarely reported. Several preclinical studies have been performed on non-solid tumors such as glioblastoma (202, 203) and lymphoblastic leukemia (204). This approach also exerted significant efficacy in the treatment of multi-drug or ICI-resistant patients (205, 206). Although RIT shows great potential, the efficacy is still limited by RT protocols in clinical practice.

Conventional fractionation RT schedules are not optimal for immune activation. Conventional fractionated RT involves delivering 2 Gy fractions per day for several weeks up to a total dose of 40–80 Gy to the targeted tumor (207). However, healthy tissues are repeatedly exposed to irradiation, leading to increased recruitment of immunosuppressive cells, including Tregs, M2 and N2, downregulation of MHC expression on cancer cells, and activation of stress response pathways that enhance tumor resistance (8). Several clinical trials have shown that this combination strategy results in all-grade AEs of ~90% and grade ≥3 AEs of 12.4% (208), respectively, with no improvement in treatment efficacy (209) or infiltration of immune cells (210). Furthermore, the decrease in absolute lymphocyte counts is more profound in conventional fractionated RT (211). For stereotactic body radiation therapy (SBRT), it delivers high doses per fraction (>8 Gy per fraction), which reduces irradiated volume and fractionation. When combined with immunotherapy, SBRT improves overall survival (OS) and response rate by increasing the recruitment of CD8^+^ T cells and expression of MHC (212, 213). These benefits were also observed with ablative doses (214). However, the cooperative effects of this combination were lost or even induced more serious AEs (215), leading to treatment suspension (216). This may attribute to exposure of healthy tissue to high doses of radiation, leading to upregulation of TREX1, which reduces the cGAS-STING pathway and weakens the antitumor ability of IFN-I (106). By contrast, low-dose radiotherapy (LDRT) delivered in a single or a few fractions (0.5–1 Gy per fraction) has shown significant immune-activating and therapeutic activity. Studies indicated that LDRT, either alone or in combination with ICIs and/or high-dose RT, promoted the conversion of an immunosuppressive TME into an immune-infiltrated one with M1, CD8^+^ T cells, Th1-polarized CD4+ T cells, and NK cells, and reduced expression of immunosuppressive cytokines such as TGF-β (217–220).

The radiation-induced toxicity to immune organs is often overlooked in clinical radiation protocols. For instance, tumor-draining lymph node irradiation has been applied in some types of HNSCC and prostate cancer to reduce the potential for tumor recurrence (221). However, this leads to radiation-induced damage to the priming of the immune system, including inhibited migration and antigen presentation of DC cells (222), prevented the priming and proliferation of CD8^+^ T cells (223), and inhibited the occurrence of the abscopal effect (224), especially at high doses (225). This can be more serious when applying conventional fractionation RT. This issue is relevant when the large volumes of vasculature and lymph node are irradiated and when the immune organs are unintentionally irradiated (226, 227).

Regarding the sequence and timing of RT and immunotherapy, preclinical studies suggest that applying immunotherapy with a narrow window after RT may achieve superior antitumor efficacy (228). Clinically, several studies have reported that prior RT followed by ipilimumab, pembrolizumab, and nivolumab for lung cancer (229), advanced urothelial cancer (230), metastatic triple-negative breast cancer (231) and non-small cell lung cancer (NSCLC) (232) was associated with enhanced abscopal effects in some cases (233). However, these observations are largely derived from retrospective or early-phase trails, and optimal sequencing remains to be validated in prospective randomized studies. Mechanistically, preclinical studies have indicated that existing RT, especially hypofractionated RT, can activate the immune system via promoting the release of DAMPs, upregulating the expression of MHC, and recruiting antitumor immune cells (234). Subsequent administration of immune-regulated drugs can alleviate immunosuppression, leading to improved systemic antitumor responses. Furthermore, preclinical evidence suggests that advanced RT treatment can minimize radiation toxicity to circulating or already infiltrated immune cells, particularly naïve T cells and CD8^+^ T cells undergoing division and proliferation (235). However, delayed immunotherapy may be less effective due to the re-establishment of an immunosuppressive TME, resulting in diminished therapeutic efficacy and potential drug resistance. This temporal dependency underscores the need for rigorously designed clinical trials to define optimal sequencing strategies across different cancer types and immunotherapy agents.

The application of dual-or even triple-targeting proteins that inhibit multiple targets to combine with RT is a current focus (236). This strategy not only prevent the tumor from escaping single drug-based therapy through other immune checkpoints (237), but also can promote immune cell infiltration and abscopal effects, and can reduce immunosuppression and fibrosis when used simultaneously, such as combined anti-PD-1 and anti-TGF-β (238), CD40 agonist and anti-CTLA-4 (239), anti-PD-1 and anti-LAG-3 (240). However, some studies have suggested that dual anti-PD1/L1 and anti-CTLA-4 (241–243), anti-PD-1 and anti-LAG3 (244) immunotherapies induce more grade 3–4 AEs and do not boost overall survival. Similarly, when combined with RT, multiple drug regiments such as anti-PD1/L1 plus anti-CTLA-4 (245, 246) induce more 3–4 AEs without enhanced efficacy. The reason for this increased toxicity is that multiple immunotherapies may remove the protective effect of immunosuppressive cells on healthy tissues damaged by radiation (237).

### 4.4 FLASH-RT provides a novel solution for improving the efficacy of RIT

Summarize the above issues, the efficacy of RIT is influenced by the radiation dose, fraction regimen, irradiated volume, treatment timing and sequence, and the overlapping toxicity. Firstly, conventional fractions cause a large irradiated volume of circulating immune cells, vasculature, and immune organs, weakening the priming of the immune system and the foundation of immunotherapy. Secondly, even the most advanced radiation technique inevitably causes radiation-induced toxicity to healthy tissue along the radiation path. This leads to the massive release of immunogenic substances from healthy tissues, resulting in the recruitment of immunosuppressive cells and re-establishment of an immunosuppressive TME that overwhelms the regulated effects of immunotherapy. Thirdly, healthy tissue and the immune system are more likely to be damaged at high doses. The antitumor immune responses decrease due to the downregulation of the cGAS-STING-IFN-I pathway and increased TGF-β release. Fourthly, the treatment duration of CONV-RT typically spans 5–8 weeks, during which immune-activating signals may become desynchronized with the timing of immunotherapy, thereby missing the optimal window for combination. Finally, dual or triple immunotherapies combined with RT lead to overlapping toxicities, limiting the tolerable doses of combination therapy.

**Figure 5:**
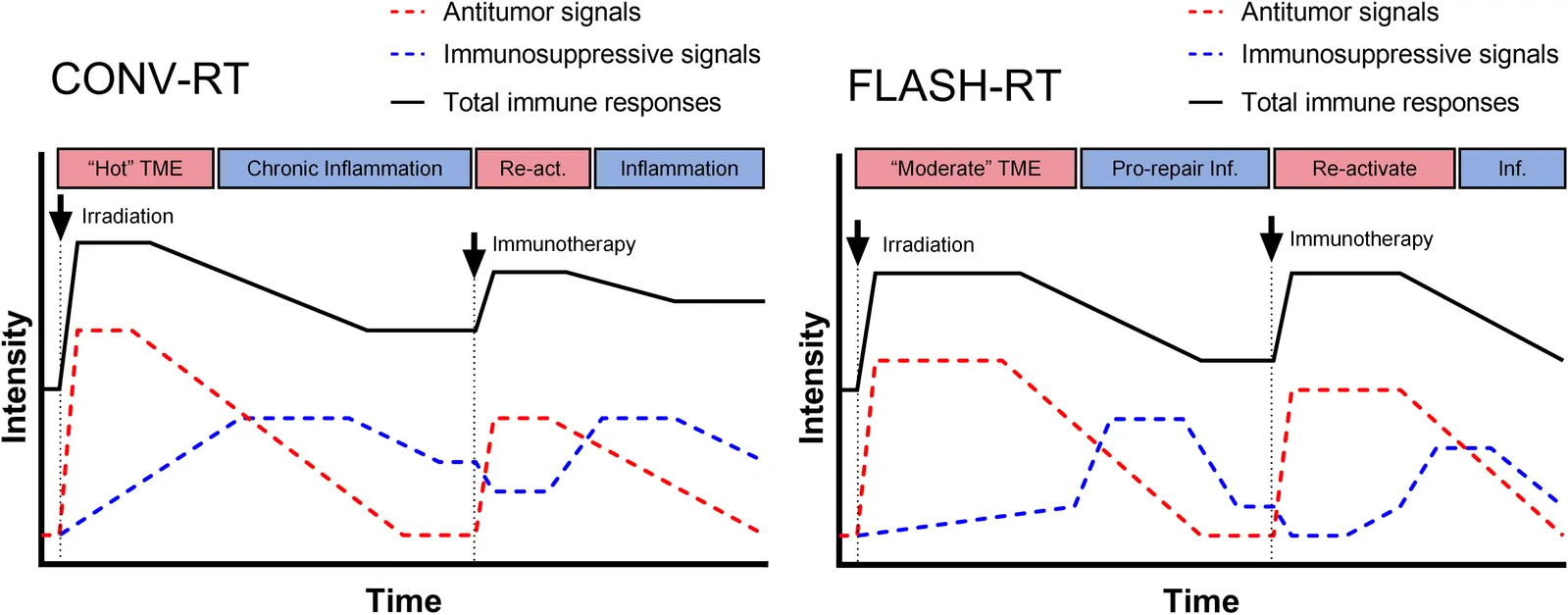
Immune response curves of FLASH-RT and CONV-RT when combined with immunotherapy. CONV-RT rapidly activates the immune system, but its high toxicity concurrently induces immunosuppressive signals that quickly re-establish an immunosuppressive TME, leading to a low response rate to immunotherapy. In contrast, FLASH-RT induces moderate antitumor immunity owing to its protective effects. Consequently, the immunosuppressive signals accumulated slowly, thereby delaying the development of an immunosuppressive TME and improving the response rate to immunotherapy.

The unique protective effect of FLASH-RT offers preferable solutions to the problems encountered in RIT. This may induce a different intensity of immune responses when combined with immunotherapy (**Figure 5**). Firstly, FLASH-RT significantly reduces irradiated volume to healthy tissue and the immune system, owing to its ultra-rapid radiation delivery in a single high-dose fraction or hypofractionated schedule, which ensures immune priming without enhanced toxicity. Secondly, the protective effect of FLASH-RT minimizes harmful immune responses triggered by healthy tissue exposure, thereby delaying the re-establishment of an immunosuppressive TME. Thirdly, even though FLASH-RT adopts high dose delivery (>10 Gy), it fails to induce high level of TREX1 and TGF-β expression, thereby preserving antitumor immune responses. Fourthly, the moderately immune-infiltrated TME induced by FLASH-RT has a durable response platform period, giving more time for immunotherapy to exert its immune regulatory roles. Finally, the lower radiation-induced toxicity of FLASH-RT widens the therapeutic window and enhances systemic immune responses when combined with multiple immunotherapies.

## 5 Translational roadmap of FLASH-RT and its combination with immunotherapy

### 5.1 Optimizing physical consistency, dosimetric accuracy, and organ-specific thresholds

The clinical translation of FLASH-RT is limited by immature dose delivery systems, deficient physical and dosimetry parameter control, and limited preclinical and clinical treatment protocols. To address these challenges, the priority is not only to ensure the linearity of dose delivery (R^2^ >0.999) and dose measurement accuracy (dose error <3-5%), but also to develop real-time dosimeters for ultra-high dose rate conditions. While increasing the dose rate is necessary, pulse structure (dose per pulse, pulse interval, pulse number, and pulse dose rate), depth-dose distribution (dose attenuation, beam energy), irradiated direction (vertical or horizontal), and irradiated field (round or square) must be systematically characterized and biologically validated *in vivo*. Moreover, physical and dosimetric standardization needs to be coupled with recognition of tissue-specific biological determinants, given that the FLASH effect is absent in certain tissues despite identical parameters. Comparative studies evaluating antitumor efficacy and healthy tissue protection across different radiation modalities (electron, photon, proton, heavy ion) and multiple tumor models are urgently needed. Furthermore, organ-specific thresholds for dosimetric parameters (total dose, dose rate, and fractionation) should be established, incorporating both positive and negative outcomes, to define the parameter boundaries of the FLASH effect. Additionally, quantification indicators of the FLASH effect such as DMF should be applied to specific organs under specific conditions. Future efforts need to establish an organ specific DMF database.

### 5.2 Combing FLASH-RT with current clinical radiation techniques

Image guided radiotherapy (IGRT) uses imaging to identify and track target tumors to enhance precision and minimize radiation exposure to healthy tissue (247). This has been applied in the treatment of a canine patient (248), enabling real-time monitoring of motion during FLASH-RT, though it cannot suspend the FLASH beam during the delivery process. Additionally, radiotheranostics–a combination of diagnostics and therapeutics–offers potential for FLASH-RT. Radiotheranostics uses radiolabeled diagnostic compounds targeting tumors, coupled with a companion imaging method, to enable visualization of tumors *in vivo* (249), achieving the aim of “treat what you see”. However, this technique is challenged by a relatively low objective response rate for bulky tumors (250). Hence, in a combination of radiotheranostics and FLASH-RT, radiotheranostics may help monitor real-time changes in tumor volume and targeted metastatic tumor, while FLASH-RT attacks the primary tumor and enhances systemic antitumor responses with lower toxicity to healthy tissue. Moreover, FLASH-RT is also compatible with hypofractionated schedules, including IMRT, VMAT, and SBRT. This combination can achieve a coherent high-dose distribution within the target volume to maximize tumor coverage (251–253), while reducing potentially large dose toxicity to healthy tissue (254). Notably, an innovative technique called spatially fractionated radiotherapy (SFRT), also has great potential in combination with FLASH-RT. SFRT modulates the dose distribution spatially with alternating high- and low-dose regions (255), rather than the average dose profiles used in CONV-RT. Similar to the FLASH effect, SFRT reduces the irradiated volume of normal tissue (256, 257), showing significant protective effects, which do not rely on local oxygen consumption (258, 259). Currently, the combination of FLASH-RT and SFRT has become a hot topic, which may integrate their advantages to obtain better protective efficacy on both spatial and temporal scales, especially to reduce the potential toxicity of the peak dose in SFRT (260). Another non-homogeneous irradiation method (half shielding), which is easier to implement, can also be combined with FLASH-RT to reduce the potential toxicity of the high dose (261).

### 5.3 Investigating mechanistic insights, large-animal verification, and tissue-dependent dosimetric parameters

Several hypotheses have been proposed to explain the FLASH effect, including oxygen depletion and ROS-associated hypothesis, mitochondria metabolism and iron metabolism hypothesis, DNA integrity hypothesis, and immune-mediated hypothesis. However, numerous studies concentrate on superficial phenomena and lack a deeper molecular analysis. Experimental models need to be extended to large animal models (rabbit, cat, dog, and minipig) and multiple organs beyond skin, lungs, brain, and intestine. Moreover, some crucial hypotheses are derived primarily from in silico or *in vitro* experiments, which need to be confirmed *in vivo* models. *In vivo* experiments also need to determine the differences in key characteristics among various organs, including oxygen concentration, antioxidant ability, mitochondria density, labile Fe content, immune status, vascular maturity, and DNA repair capacity, which may explain various or even opposite outcomes across preclinical studies and facilitate the determination of clinical indications. Additionally, the role of the RLR-MAVS pathway, microbiota-derived products, healthy stem cells, and tumor stem cells under FLASH-RT remains unclear. The different structures and mechanics between tumor and healthy tissues can also influence the FLASH effect (262).

### 5.4 Clarifying FLASH-RT-driven cellular and molecular remodeling of the TME

FLASH-RT remodels a moderately immune-infiltrated TME distinct from that induced by CONV-RT. Such TME remodeling sustains a relative long-term antitumor immunity, alleviates chronic inflammation, and prevents the rapid re-establishment of an immunosuppressive TME. Notably, these immunomodulatory effects are supported only by limited preclinical models, with most studies focusing on determining the ratio of CD8^+^/CD4^+^ T cells and the number of CD8^+^ T, CD4^+^ T, Treg, NK, M1, and M2 cells within the TME. Accordingly, future investigations should move beyond simple immune cell enumeration toward standardized, multi-model comparisons that clarify the anti/protumor roles, distribution among tumor and healthy tissue, and the crosstalk mediated by alterations in key cytokines, immune regulators, and cell surface molecules among other cells in the TME, such as DCs, TAFs, TANs, and MDSCs. Furthermore, infiltration and spatial distribution of immune cells within the TME under varying acidic and oxygen concentrations remain poorly characterized. Memory-related and systemic immune responses (such as the abscopal effect and anti-metastasis immunity) still require validation in large-animal models or early-phase clinical trials. Moreover, immunological differences between single-fraction and hypo-fractionated FLASH-RT are largely underexplored and warrant systematic evaluation (263). Besides, low-dose FLASH-RT, re-irradiation regimens, and gut microbiota-modulated immune regulation constitute promising yet underexplored research avenues that may broaden future clinical application prospects.

### 5.5 Elucidating optimal combination strategies, treatment sequence, and timing remain

Although FLASH-RT combined with immunotherapy exerts synergistic effects against drug-resistant tumors, the combination still faces translational challenges. Current research is largely restricted to several animal and tumor models, with immune characterization focusing predominantly on CD8^+^/CD4^+^ T cells and TAMs. Additional studies are needed to explore the roles of other immune cells when combined with immunotherapy, such as TAFs, TANs, NKs, MDSCs, and DCs. Moreover, the involvement of cytokines like IL-12 (264), CXCL-9, CXCL-10, CXCL-11 (265), which drive early stage immune responses, and some immunosuppressive molecules such as TGF-β, IL-2, IL-4, IL-6, IL-8, IL-10, VEGF, G-CSF, CCL-2, CCL-7, CXCL-12 requires further investigation (266–268). Some novel immune checkpoints, including LAG-3 and TIM-3, are still lacking in clinical studies and could be the underlying approaches in combination with FLASH-RT (269).

Furthermore, the combination of FLASH-RT and ACTs has been poorly studied. This combination has great potential for reducing the radiation-induced toxicity to injected CAR-T cells or CAR-NK cells, enhancing lymphocytic infiltration, and attenuating the establishment of immunosuppression. Additionally, integrating FLASH-RT with tumor vaccines, oncolytic viruses, and immune agonists such as anti-CD40, anti-CD47, and CD169 agonists (270, 271), as well as TLR9 and TLR7 agonists (272, 273), may elicit stronger systemic antitumor immune responses while inducing lower radiation-induced toxicity.

Notably, critical knowledge gaps persist regarding optimal treatment scheduling. While preclinical study suggest FLASH-RT establishes a short-term pro-repair inflammatory window (around Day 10 post irradiation). If immunotherapy is administered in this period, the pro-repair effect may disappear, leading to loss of its protective and synergistic effect. Current studies show administering immunotherapy on Day 5, 7, 10, 13 after FLASH-RT yields beneficial effects, while Day -1, 0, 2 shows no improvement. Hence, future studies are required to identify the time point and duration of this therapeutic period, and to assess whether adding immunotherapy during this period will elicits any new effects. Moreover, the optimal sequencing of FLASH-RT and immunotherapy remains unclear.

Towards clinical translation, inconsistencies regarding the dose delivery system, physical and dosimetric parameters across preclinical studies need to be fully resolved. Technical barriers including the inability to treat deep-seated tumors and the lack of clinically feasible real-time dosimetric monitoring should be resolved. Early-phase clinical trials need a breakthrough from 0 to 1 in evaluating the safety and efficacy of combining FLASH-RT and immunotherapy, particularly for some immunosensitive tumors. These trials should incorporate suitable immune-related endpoints to better determine the immunomodulatory effects of FLASH-RT. Furthermore, patient selection should consider the tumor hypoxia status, TME characteristics, and systemic immune function, which can better identify patients most likely to benefit from the combination of FLASH-RT plus immunotherapy.

## 6 Conclusion

In summary, FLASH-RT holds the potential for combination with immunotherapy through its unique ability to induce a moderately immune-infiltrated TME. However, future research is needed to address the physical, dosimetric, and biological challenges associated with such combinations. With the rapid evolution and improvement of FLASH-RT, this combination strategy is expected to achieve superior therapeutic efficacy in the clinical treatment of tumors.

## Glossary

FLASH-RT: ultra-high dose rate radiotherapy
CONV-RT: conventional radiotherapy
RIT: radioimmunotherapy
RT: radiotherapy
IMRT: intensity-modulated radiation therapy
VMAT: volumetric-modulated arc therapy
IGRT: image-guided radiation therapy
SBRT: stereotactic body radiation therapy
ICI: immune checkpoint inhibitor
ACT: adoptive cell therapy
TME: tumor microenvironment
ROS: reactive oxygen species
PD-1: programmed death-1
LET: linear energy transfer
e-FLASH: electron FLASH
p-FLASH: proton FLASH
AEs: adverse events
X-ray FLASH: x-FLASH
SGRT: surface guided radiotherapy
SFRT: spatially fractionated radiotherapy
DAMP: damage-associated molecular pattern
TAAs: tumor-associated antigens
Tregs: regulatory T cells
TAMs: tumor-associated macrophages
TANs: tumor-associated neutrophiles
DCs: dendritic cells
NKs: natural killer cells
PD-L1: anti-programmed death ligand-1
CTLA-4: cytotoxic T lymphocyte-associated antigen-4
TGF-β: transforming growth factor-β
IL: interleukin
IFN-I: interferon-I
TNF-α: tumor necrosis factor-α
VEGF: vascular endothelial growth factor
RNS: reactive nitrogen species
SOD: superoxide dismutase
OXPHOS: oxidative phosphorylation
GPX4: glutathione peroxidase 4
dsDNA: double-stranded DNA
cGAS-STING: cyclic GMP-AMP synthase-stimulator of interferon genes
IRF-3: interferon regulatory factor 3
NF-κB: nuclear factor kappa-B
PAMP: pathogen-associated molecular pattern
ssDNA: single-stranded DNA
EMT: epithelial–mesenchymal transition
BER: base excision repair
TREX1: three prime repair exonuclease 1
mtDNA: mitochondrial DNA
Drp1: Dynamin-1-like protein
RIG-I: retinoic acid-inducible gene I
RLR: retinoic acid-inducible gene I-like receptor
MDA5: melanoma differentiation associated gene 5
LGP2: laboratory of genetics and physiology 2
MAVS: mitochondrial antiviral signaling protein
TBK1: TANK-binding kinase 1
IKK: inhibitor of kappa B kinase
ERV: endogenous retrovirus
T-ALL: T-cell acute lymphoblastic leukemia
CAMs: cell adhesion molecules
M2: tumor-promoting macrophages
G-CSF: granulocyte-colony stimulating factor
GM-CSF: granulocyte-macrophage colony stimulating factor
CSCs: cancer stem cells
TAFs: tumor-associated fibroblasts
HMGB1: high mobility group box protein 1
MHC: major histocompatibility complex
MDSCs: myeloid-derived suppressor cells
eNOS: endothelial nitric oxide synthase
NO: nitric oxide
MLC: myosin light chain
APCs: antigen-presenting cells
TIM-3: mucin domain-containing protein 3
LAG-3: lymphocyte activation gene
TIGIT: T cell immune receptor with Ig and ITIM domains
M1: tumor-killing macrophages
N1: antitumorigenic neutrophils
N2: protumorigenic neutrophils
CTL: cytotoxic T lymphocytes
CAR-T: chimeric antigen receptor T cells
MCP: monocyte chemotactic protein-1
TLR: toll-like Receptor
SA-βgal: senescence-associated β-galactosidase
PPAPγ: peroxisome proliferator-activated receptor-γ.
